# Comparison of Ondansetron and Meperidine for Treatment of Postoperative Shivering: A Randomized Controlled Clinical Trial

**DOI:** 10.5812/ircmj.13079

**Published:** 2014-08-05

**Authors:** Alireza Mahoori, Heydar Noroozinia, Ebrahim Hasani, Maryam Soltanahmadi

**Affiliations:** 1 Department of Anesthesiology, Imam Khomeini Training Hospital, Urmia University of Medical Sciences, Urmia, IR Iran

**Keywords:** Anesthetics, Adverse Effects, Meperidine, Therapeutics, Ondansetron, Shivering

## Abstract

**Background:**

The involved neurotransmitter pathways in the postoperative shivering (POS) are poorly understood. Recently, 5-hydroxytryptamine 3 (5-HT3) receptor antagonists have been reported to prevent POS. We investigated the effect of ondansetron, a 5-HT3 antagonist that is used to treat postoperative nausea and vomiting, on shivering.

**Objectives:**

This study aimed to compare the efficacy of ondansetron and meperidine in the treatment of shivering after general anesthesia.

**Patients and Methods:**

In this double-blinded randomized clinical trial, 83 patients (age range, 18-60 years) who had shivering after general anesthesia were randomly allocated to any of these three groups: Group A, (number = 27) received 4 mg of intravenous ondansetron, Group B, (number = 27) received 8 mg of intravenous ondansetron, and Group C, (number = 29) received 0.4 mg/kg of intravenous meperidine at recovery room. The surface temperatures and the incidence as well as intensity of shivering were recorded.

**Results:**

Shivering was controlled in 16 patients (59%) in Group A, 22 (81%) in Group B, and 25 (86%) in Group C (P = 0.01). Within each group, there were no significant differences among the surface temperature in recovery room. Patients in groups A and B had significantly lower incidence of nausea and vomiting than group C (P = 0.01).

**Conclusions:**

Ondansetron and meperidine have similar effects on shivering. We concluded that 8 mg of intravenous ondansetron can control shivering and this is the dose of choice, especially in patients with POS with nausea and vomiting.

## 1. Background

The incidence of postoperative shivering (POS) was reported to be 40%; however, nowadays it appears to be less frequent as more patients are kept normothermic during perioperative period. Shivering is a serious complication that increases oxygen consumption by about 100% in proportion to intraoperative heat loss (1). Shivering causes ocular and intracranial hypertension as well as pain due to tension of surgical incision site. The most important risk factors for shivering are young age and hypothermia. Unfortunately, the main cause of shivering is still unknown. One of the therapeutic methods for shivering is superficial heating (surface temperature); however, the skin surface contributes to only 20% of shivering control and it can be useful and applicable only in the case of slight hypothermia and core temperature > 35℃ (2). POS can also be treated with a variety of drugs, including intravenous clonidine (75 µg), ketanserin (10 mg), tramadol, physostigmine (0.04 mg/kg), nefopam (0.15 mg/kg), dexmedetomidine, and magnesium sulfate (30 mg/kg). Meperidine is used to treat shivering but has some side effects such as nausea, vomiting, and respiratory depression (1, 3-5).

Neurotransmitters regulate the body temperature and each of the abovementioned drugs has a special effect on these neurotransmitters. One of these regulatory neurotransmitters is 5-hydroxytryptamine (5-HT) or serotonin. Serotonin is a biological amine that acts as a neurotransmitter in the brain and spinal cord (6). Recently, there has been a considerable attention toward serotonin receptor antagonists in the prevention of shivering (7). In animal models, direct intraventricular injections of 5-HT influences body temperature and shivering. In fact, tramadol, which inhibits 5-HT reuptake, and ketanserin, a 5-HT2 antagonist, inhibit established POS (1). Ondansetron is a specific 5-HT3 antagonist and seems to be effective in the control of temperature and POS. In previous studies, mostly preventative effects of ondansetron and 5-HT2 antagonists on POS has been studied, and useful results were obtained (8, 9).

## 2. Objectives

For treatment of POS, mostly narcotics, particularly meperidine, are used that may have some side effects such as respiratory depression, nausea, and vomiting. Therefore, we evaluated the effect of two different doses of ondansetron on POS and compared the results with that of meperidine, which is frequently used in control of POS.

## 3. Patients and Methods

The Scientific and Ethical Review Board of Urmia University of Medical Sciences, Urmia, Iran, approved the study protocol (Code 91/2/18/4512). All patients who were admitted to Urmia, Imam Khomeini Teaching Hospital signed a written informed consent prior to participation in the study. In this double-blinded randomized clinical trial, 90 patients undergoing routine general, orthopedic, and plastic surgery who developed shivering in the recovery room were studied from June 2012 to January 2013. Included patients had ASA physical status I or II, aged 18 to 60 year, and weighed 60 to 80 kg. Exclusion criteria were a febrile illness, history of lung disease, allergy to ondansetron or meperidine, history of seizure, use of vasoconstrictors, high intracranial pressure, and history of drug abuse. Routine care for POS such as using covering blanket, and using oxygen via the facemask was performed. Shivering was graded with a scale (Table 1) similar to the one that was validated by Crossley and Mahajan (10).

Only patients who developed grade three or four shivering for at least three minute were included. Regarding the 43% difference between groups in previous similar studies, power of 80%, and coefficient α of 0.05, sample size was calculated at 30 patients for each group. Using computer-assisted simple randomization, patients were randomly allocated to one of the following groups:

- (4 mg of ondansetron),
- (8 mg of ondansetron), and
- (0.4 mg/kg of meperidine).

All preloading fluids and drugs were used at room temperature. Study medications were prepared at the same volume by technicians who were not involved in the study.

The anesthesiologist, who was unaware of the patients’ assigned group and treatments, measured the elapsed time from treatment to the time of shivering cessation. If shivering did not cease after ten minute, the treatment was regarded as ineffective. In all patients, surface temperature was recorded at axilla by a skin thermometer. Kolmogorov-Smirnov test showed normal distribution of variables. One-way ANOVA was used to analyze differences among the study groups. Incidence of shivering was analyzed by using χ2 test. Data were expressed as mean ± SD and number (percentage). P value < 0.05 was considered as statistically significant.

**Table 1.** Shivering Rating System

Grade	
**0**	Nonshivering
**1**	Piloerection or peripheral vasoconstriction, but no visible shivering
**2**	Muscular activity only in one muscle group
**3**	Muscular activity in more than one muscle group
**4**	Shivering involving the whole body

## 4. Results

Three patients from group A, three from B, and one from C were excluded from the study due to receiving excessive analgesic and other drugs in the postanesthesia care unit and the possibility of having incorrect results. Totally 83 patients who had POS with the intensity of grade three or four were evaluated. Demographic data including age, sex, and weight, surface temperature, and intensity of shivering showed no difference among the study groups (Table 2).

The rate of response to treatment (shivering cessation after ten minute) was 59%, 81%, and 86% for groups A, B, and C, respectively (P = 0.01, Table 3). Incidence of nausea and vomiting were 11%, 0%, and 34% for groups A, B, and C, respectively (P = 0.01, Table 3).

**Table 2.** Demographic Data and Patient Characteristics ^a^

Variable	Group A (n = 27), 4 mg of Ondansetron	Group B (n = 27), 8 mg of Ondansetron	Group C (n = 29), 0.4 mg/kg of Meperidine	P Value
**Sex, M/F**	11/16	10/17	11/18	0.72
**Age, y**	32 ± 2	32 ± 8	32 ± 10	0.65
**Weight, kg**	66 ± 7	70 ± 9	68 ± 10	0.44
**Intensity of Shivering**	3.5 ± 0.5	3.4 ± 0.5	3.6 ± 0.4	0.39
**Surface Temperature, ℃ **	36.8 ± 0.1	36.8 ± 0.2	36.8 ± 0.1	0.94

^a^ Data are presented as number or mean ± SD.

**Table 3.** Response to Treatment and the Incidence of Nausea and Vomiting ^a^

Variable	Group A (n = 27), 4 mg of Ondansetron	Group B (n = 27), 8 mg of Ondansetron	Group C (n = 29), 0.4 mg/kg of Meperidine
**Response to Treatment**	16 (59)	22 (81) ^b^	25 (86) ^b^
**Nausea and Vomiting **	3 (11) ^c^	0 (0) ^c^	10 (34)

^a^ Data are presented as No. (%).

^b^ P ˂ 0.05 versus group A.

^c^ P ˂ 0.05 versus the group C.

## 5. Discussion

Treatment of POS is an important aspect of patient care since it may cause serious complications such as sympathoadrenal stimulation, increased oxygen consumption, and carbon dioxide production. In this study, 22 patients (81%) who had received 8 mg of ondansetron responded to treatment and their shivering was controlled. On the other hand, shivering ceased in only 16 patients (59%) who had received 4 mg of ondansetron. Therefore, ondansetron with the dose of 8 mg is as effective as 25 mg meperidine in controlling POS and there was no significant difference between them in this respect. Specific inhibition of the serotonin system might have produced a dose-dependent reduction in POS.

Serotonin inhibition may have a direct effect on shivering; however, neurotransmitter systems are also effective in control of shivering. An inhibitory effect on the 5-HT3 receptor might result from a generalized thermoregulatory inhibition at the level of the hypothalamus, where the main thermoregulatory control occurs (1). As mentioned earlier, due to redistribution of heat from center to the peripheral parts and body surface, the core temperature reduces by 1℃ after induction of anesthesia and at the same time, the temperature of fingers increases slightly (11).

In this study, we did not measure the temperature during the operation but surface temperature was measured in recovery and no significant difference was found between the groups receiving meperidine and ondansetron. It seems that ondansetron does its antishivering effects through a central mechanism; in fact, the drug has no effect on hemodynamic variables and it seems unlikely to exert its effects through the peripheral receptors (8). Contrary to some other drugs, which are used for treatment of POS, ondansetron has no effect on cardiovascular systems. For example, clonidine may cause hypotension or sedation and physostigmine can decrease heart rate and blood pressure; therefore, oxygen consumption may increase in patients with coronary artery disease (12). On the other hand, ondansetron effectively reduces postoperative nausea and vomiting. In various studies, some other 5-HT antagonists such as tramadol were used for prevention or treatment of shivering and in most of them, reduction in sweating, vasoconstriction, and shivering threshold were reported (9, 13, 14). Granisetron, which is also a new 5-HT3 inhibitor and is mostly used to control of postoperative nausea and vomiting, is recently used for prevention of POS (15, 16). These studies indicate the role of serotonin receptors in thermoregulation at different levels. Nevertheless, some studies have reported that ondansetron did not change the threshold of shivering and probably reduces shivering thorough the central effects and ondansetron does not decrease the incidence or severity of shivering in women undergoing elective cesarean delivery (17, 18). Although 0.4 mg/kg of meperidine rarely causes unwanted cardiovascular effects, it may cause respiratory depression, particularly when used with other opioids. On the other hand, it increases the chance of postoperative nausea and vomiting. Ondansetron reduces POS, nausea, and vomiting simultaneously.

Some studies have worked mainly on the preventative effects of ondansetron on POS. Powell et al. compared the effect of 4 mg and 8 mg ondansetron with placebo in preventing POS in surgical procedures. They reported that administration of 8 mg ondansetron before the induction of anesthesia could reduce the incidence of POS in adults without effecting the core to peripheral redistribution of temperature (8). Some authors have compared the effect of ondansetron with meperidine in preventing POS and reported similar antishivering effects for meperidine and ondansetron (19-21).

One of the limitations to the present study was that we could not measure the core temperature during the preoperative period, and we could not find another checklist for assessment of shivering. In previous studies, mostly preventative effects of ondansetron and 5-HT2 antagonists on POS has been studied; however, we evaluated the effect of two different doses of ondansetron on treatment of POS and compared the results with that of meperidine, which might be the strength of our study.

The results of this study were completely consistent with these studies. In our study, ondansetron and meperidine were used for treatment of shivering and no additional medication was administered as a preventative drug. We demonstrated that 4 mg of ondansetron had little effect on treatment of shivering and the effective dose is 8 mg. In conclusion, both 8 mg ondansetron and 0.4 mg/kg meperidine effectively treated patients with POS; however, selecting 8 mg ondansetron, especially in patients with postoperative nausea, vomiting, and shivering would be an ideal choice.

## References

[1] Sessler DL (2010). Tempratture Regulation and Monitoring..

[2] Giesbrecht GG, Ducharme MB, McGuire JP (1994). Comparison of forced-air patient warming systems for perioperative use.. Anesthesiology..

[3] Sajedi P, Nazem Alroaya B (2006). Comparing the effectiveness of antishivering action of meperidine alfentanil, sufentanil, fentanyl and tramadol after general anesthesia.. Shiraz E-Medical Journal..

[4] Davoudi M, Mousavi-Bahar SH, Farhanchi A (2007). Intrathecal meperidine for prevention of shivering during transurethral resection of prostate.. Urol J..

[5] Talakoub R, Noori Meshkat S (2006). Tramadol versus meperidine in the treatment of shivering during spinal anesthesia in cesarean section.. J Res Med Sci..

[6] Wilson AJ, Diemunsch P, Lindeque BG, Scheinin H, Helbo-Hansen HS, Kroeks MV (1996). Single-dose i.v. granisetron in the prevention of postoperative nausea and vomiting.. Br J Anaesth..

[7] Hindle AT (1994). Recent developments in the physiology and pharmacology of 5-hydroxytryptamine.. Br J Anaesth..

[8] Powell RM, Buggy DJ (2000). Ondansetron given before induction of anesthesia reduces shivering after general anesthesia.. Anesth Analg..

[9] Mohta M, Kumari N, Tyagi A, Sethi AK, Agarwal D, Singh M (2009). Tramadol for prevention of postanaesthetic shivering: a randomised double-blind comparison with pethidine.. Anaesthesia..

[10] Crossley AW, Mahajan RP (1994). The intensity of postoperative shivering is unrelated to axillary temperature.. Anaesthesia..

[11] Ikeda T, Sessler DI, Kikura M, Kazama T, Ikeda K, Sato S (1999). Less core hypothermia when anesthesia is induced with inhaled sevoflurane than with intravenous propofol.. Anesth Analg..

[12] Horn EP, Standl T, Sessler DI, von Knobelsdorff G, Buchs C, Schulte am Esch J (1998). Physostigmine prevents postanesthetic shivering as does meperidine or clonidine.. Anesthesiology..

[13] Heid F, Grimm U, Roth W, Piepho T, Kerz T, Jage J (2008). Intraoperative tramadol reduces shivering but not pain after remifentanil-isoflurane general anaesthesia. A placebo-controlled, double-blind trial.. Eur J Anaesthesiol..

[14] Trekova NA, Buniatian AA, Zolicheva N (2004). [Tramadol hydrochloride in the treatment of postoperative shivering].. Anesteziol Reanimatol..

[15] Sagir O, Gulhas N, Toprak H, Yucel A, Begec Z, Ersoy O (2007). Control of shivering during regional anaesthesia: prophylactic ketamine and granisetron.. Acta Anaesthesiol Scand..

[16] Sajedi P, Yaraghi A, Moseli HA (2008). Efficacy of granisetron in preventing postanesthetic shivering.. Acta Anaesthesiol Taiwan..

[17] Komatsu R, Orhan-Sungur M, In J, Podranski T, Bouillon T, Lauber R (2006). Ondansetron does not reduce the shivering threshold in healthy volunteers.. Br J Anaesth..

[18] Browning RM, Fellingham WH, O'Loughlin EJ, Brown NA, Paech MJ (2013). Prophylactic ondansetron does not prevent shivering or decrease shivering severity during cesarean delivery under combined spinal epidural anesthesia: a randomized trial.. Reg Anesth Pain Med..

[19] Kelsaka E, Baris S, Karakaya D, Sarihasan B (2006). Comparison of ondansetron and meperidine for prevention of shivering in patients undergoing spinal anesthesia.. Reg Anesth Pain Med..

[20] Abdollahi MH, Forouzannia SK, Bagherinasab M, Barzegar K, Fekri A, Sarebanhassanabadi M (2012). The effect of ondansetron and meperedin on preventing shivering after off-pump coronary artery bypass graft.. Acta Med Iran..

[21] Asl ME, Isazadefar K, Mohammadian A, Khoshbaten M (2011). Ondansetron and meperidine prevent postoperative shivering after general anesthesia.. Middle East J Anesthesiol..

